# Global prevalence estimates of three chronic musculoskeletal conditions: club foot, juvenile idiopathic arthritis and juvenile systemic lupus erythematosus

**DOI:** 10.1186/s12969-020-00443-8

**Published:** 2020-06-12

**Authors:** Mona Dave, Judith Rankin, Mark Pearce, Helen E. Foster

**Affiliations:** Population Health Sciences Institute, Newcastle University, The Medical School, Framlington Place, Newcastle upon Tyne, Tyne and Wear NE24HH UK

**Keywords:** Global Health, Prevalence, Burden, Musculoskeletal, JIA, JSLE, Clubfoot

## Abstract

**Background:**

Musculoskeletal (MSK) conditions are a major source of morbidity and disability. There is a lack of global comparable data on the burden of MSK conditions in children and young people. Our aim was to estimate the global prevalence of three MSK conditions - Talipes Equinovarus (Clubfoot), Juvenile Idiopathic Arthritis (JIA) and Juvenile Systemic Lupus Erythematosus (JSLE).

**Methods:**

Using reported prevalence rates, age-stratified population data within the World Bank Data Bank in 2017 and United Nations country classification, we estimated the prevalence of these MSK conditions in < 5 year olds (clubfoot) and < 16 year olds (JIA and JSLE) across the world.

**Results:**

We estimated that in 2017, there were ~ 675,061 < 5 year olds with clubfoot among 675,100,000 < 5 year olds, ~ 2,069,246 < 16 year olds with JIA and ~ 206,931 < 16 year olds with JSLE per 2,069,000,000 < 16 year olds, totalling ~ 2,951,238 with one of these conditions. Disease prevalence was greatest in Asia (South Asia), followed by Africa, Americas, Europe and Oceania.

**Conclusions:**

An estimated 3 million children and young people globally are currently living with either clubfoot, JIA or JSLE; many in Asia and Africa. Further work is needed urgently to engage with global stakeholders to work together to improve access to effective care for the many who are affected and reduce the otherwise adverse lifelong impact on their health, quality of life and the impact on society.

## Background

### The importance of musculoskeletal conditions in children and young people

Musculoskeletal (MSK) conditions are a leading cause of disability and morbidity and encompass conditions within rheumatology and orthopaedics as well as being the consequences of trauma and obesity. MSK conditions in adults are a major contributor to the global non-communicable diseases (NCDs) burden [1] and a priority for the global community to address [2–4] in the World Health Organisation (WHO) strategy for Universal Health Coverage [5]. Compared to 43.9% in 1990, NCDs accounted for 61.4% of global disability adjusted life years (DALYs) in the recent Global Burden of Disease report [1, 4] with the shift in disease burden from communicable to NCDs being more apparent in Low and Middle Resource Income countries (LRIC, MRIC).

What is less clear, however, is the global burden of MSK conditions for individuals under the age of 18 years; there is a paucity of population data, especially in the most populated parts of the world [4]. In the UK, it is estimated that 1 in 8 children are affected by MSK conditions [6] - many are self-limiting or related to trauma although there are also serious MSK conditions with potential to be life threatening or cause severe disability if untreated. With advances in treatments for many MSK diseases, it is more important than ever to address inequity in access to care and to include children (who represent at least 20% of the global population, [7]) in strategies to reduce MSK disability across the life course.

The purpose of our work is to inform the ‘bigger picture’ and to estimate global prevalence of three MSK conditions in children and young people using population based methods [7]. This is a pragmatic approach in the absence of robust global population studies and provides a novel perspective to inform dialogue to address unmet need.

## Methods

### MSK conditions

The chosen MSK conditions are Talipes Equinovarus (‘Clubfoot’), Juvenile Idiopathic Arthritis (JIA) and Juvenile Systemic Lupus Erythematosus (JSLE). The rationale for their inclusion was the evidence base of estimated prevalence rates, the importance of early diagnosis, and the importance of access to the right care to optimise clinical outcomes.

Clubfoot can affect one or both feet, commonly occurring in isolation, but may also be associated with other congenital anomalies [8, 9]. Clubfoot in most cases can be effectively treated using the validated Ponseti method [10, 11] but without appropriate treatment can result in pain, disability and social handicap [12]. In this report, we use the term ‘clubfoot’ to represent isolated structural clubfoot. We use the term ‘prevalence’ in the under 5 year old age group, rather than incidence, as we do not know how many affected foetuses may have been ‘lost’ through early miscarriage and the condition is congenital. Prevalence rates of clubfoot vary between countries and racial groups; for this study we used an estimated prevalence rate of 1 in 1000 children based on reports from Europe [8] and North America [9].

JIA is a heterogeneous group of diseases with markedly improved clinical outcomes since the introduction of highly effective immunosuppressives and specialist care [13, 14]. Prevalence studies are predominantly reported from Caucasian populations in Europe, North America and Australia with further evidence of variance in the prevalence of JIA subtypes in different ethnic groups and countries [15–20]; to our knowledge there are no population based studies from Africa or Asia. Such variation in prevalence rates reflect historical challenges in disease classification, ascertainment and referral biases but may also reflect true disease variation within different ethnic populations [15]. For the purpose of our work, we have used the estimated prevalence of 1 in 1000 based on the pooled prevalence data [15] and have not attempted to estimate JIA subtypes, or address variation by age, gender or ethnicity.

JSLE is a multisystem disease with serious, potentially life threatening organ involvement [21–24] and reported to be more common and severe in non-Caucasians with worse clinical outcomes [21, 23, 25–27]; to our knowledge there are no population based prevalence studies in Asia or Africa. For the purpose of this study, we have assumed a prevalence of 10 in 100,000 based on the United States (US) Medicaid programme covering a multi-racial population [22]; we have not attempted to address variation in prevalence estimates by age, gender or ethnicity.

### Data sources

The World Bank Group (WBG) [7] online Databank provides free access to a range of datasets related to global development which can be downloaded in a range of formats. For our study, population data for 2017 were downloaded from the ‘Population estimates and projections database’ within the WBG Databank for every country available within the database (*n* = 217) and stratified by age in single years from 0 to 15 years for males and females separately. The total population of countries (all ages) was extracted similarly. Information on geographic regions and sub-regions for classification of countries, was taken from the United Nations (UN) Statistics Division [28].

### Data management

Each country was manually assigned to its corresponding region and sub-region as classified by the UN [28] with the exception of Cyprus and Turkey which were classified as being in - Asia, Western Asia - by the UN [28] - but were re-classified to - Europe, Southern Europe - for the purposes of our analysis. Grouping of countries within their respective regions and sub-regions is shown in Additional file 1: Appendix A. Countries within the WBG Databank, differ from those within the UN Statistics Division. Countries with missing population data or a missing UN regional classification were excluded from our datasets and not included in any further analysis.

### Estimation of prevalence rates

The population of < 5 years olds was calculated by summing the population within each age band from 0 to 4 years for males and females separately. These were then summed to give the total population of all children < 5 years old by country, region and sub-region. The same process was undertaken to calculate the population of < 16 year olds using the population within each age band from 0 to 15 years. Estimated prevalences were calculated for 2017 using the reported prevalence rates of 1/1000 for clubfoot, 1/1000 for JIA and 10/100,000 for JSLE and the 2017 population of < 5 year olds (for clubfoot) and < 16 year olds (for JIA and JSLE) by country, region and sub-region using the following equation: *Estimated prevalence of MSK condition = Reported prevalence rate X Population.* Data management and analysis were undertaken within Microsoft Excel and Stata version 14.

## Results

**Fig. 1:**
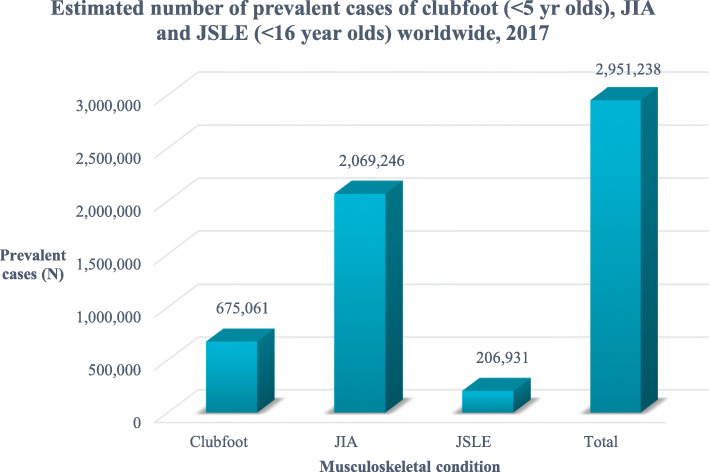
Total estimated number of cases of clubfoot, JIA and JSLE across all countries included in analysis, 2017. The totals here are the sums of individual country data and will therefore be slightly different to the total when calculated by summing region data or sub-region data in subsequent figures due to rounding error

**Fig. 2:**
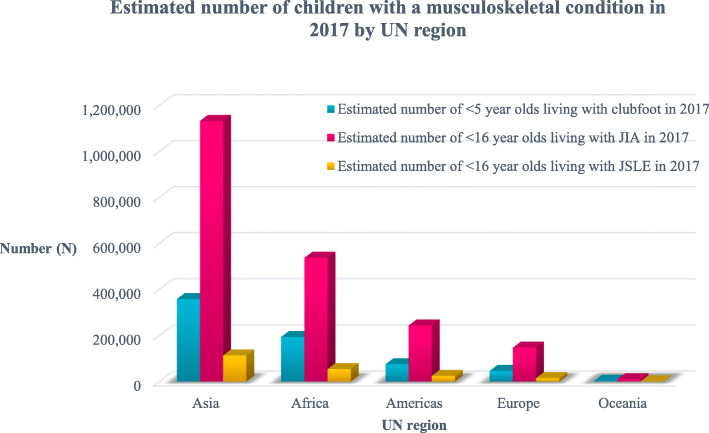
Estimated number of children in 2017 with clubfoot, JIA and JSLE by UN region

**Fig. 3:**
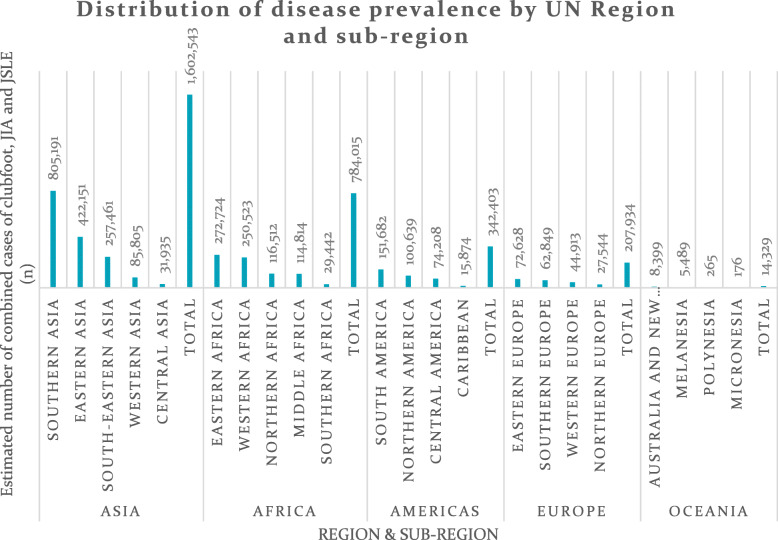
Estimated musculoskeletal conditions prevalence in 2017 by UN region and sub-region

Table 1 summarises the data available in 2017 that were used to establish the global prevalences of the selected MSK conditions. Over 200 countries and almost 7.5 billion people (with just over 2 billion < 16 year olds) were included in the analysis. Across all included countries, we estimated over 675,000 children < 5 years of age living with clubfoot, over 2 million < 16 year olds with JIA and over 200,000 with JSLE. Overall, we estimated nearly 3 million children were living with one of these conditions in 2017 (Fig. 1) with the data stratified by UN region and sub-region (Figs. 2 and 3 respectfully). Further analysis described the relative prevalence in different countries within the UN regions and sub-regions (Additional file 1: Appendix B). Asia (in particular Southern and Eastern Asia) had the highest estimated numbers of individuals with clubfoot, JIA and JSLE followed by Africa, the Americas and Europe, with Oceania having the lowest estimated numbers for all three conditions. The distribution across each UN region can be seen in Fig. 2. Most clubfoot burden was concentrated across three sub-regions: Southern Asia, Eastern Asia and Eastern Africa, whilst 51% of both JIA burden and JSLE burden was concentrated across these same regions. When further stratifying analysis by sub-region, we found Southern Asia had the highest estimated case numbers for clubfoot (176,462); 26% of clubfoot burden was distributed within this sub-region. This was followed by Eastern Asia (95,085 estimated cases, 14%) and Eastern Africa (66,158 estimated cases, 10%). Southern Asia also had the highest estimated numbers of JIA cases (571,572) with 28% of disease burden distributed across this sub-region. This was followed by Eastern Asia (297,333 estimated cases, 14%) and Eastern Africa (187,787 estimated cases, 9%). A similar pattern was seen for JSLE with 57,157 estimated cases in Southern Asia, representing 28% of disease burden, followed by Eastern Asia (29,733 estimated cases, 14%) and Eastern Africa (18,779 estimated cases, 9%). The data are presented for each country (Additional file 1: Appendix C). In absolute terms, the countries with highest numbers of CYP with the exemplar conditions reflect the overall population sizes; in descending order these are India, China, Nigeria, Pakistan, Indonesia and the United States.

**Table 1:** Summary of the World Bank Group Data (2017) used in the analysis

Variable	N
Countries listed in World Bank Group Data	217
Countries with missing UN region data	2
Countries with missing World Bank population data	23
Countries excluded from estimate calculations (data not available)	25
Countries included in analysis	192
Regions listed in UN Statistics Division	5
Sub-regions listed in UN Statistics Division	22
Total population < 5 years old across all countries with population data Male: Female	6.75 × 10^8 (675,100,000) 1.07:1.00
Total population < 16 years old across all countries with population data Male: Female	2.07 × 10^9 (2,069,000,000) 1.07:1.00
Total population across all countries included in analysis	7.49 × 10^9 (7,494,205,912)
Proportion of total population < 5 years old	9.01%
Proportion of population < 16 years old	27.61%

## Discussion

Our work has taken a pragmatic approach to estimate global prevalence of three MSK conditions and using WBG population data from 2017, suggests that nearly 3 million individuals have at least one of clubfoot, JIA or JSLE. Given that our analysis is limited to three conditions and we have not included trauma, then clearly the true MSK prevalence around the world is much higher. If we were to use our pragmatic model with the 2017 estimated global population of 2.07 × 10^9^ young people aged under 16 years and a reported prevalence 1 in 8 [6] having an MSK presentation of one kind or another, then we estimate nearly 26 million individuals are likely to be affected.

This work aims to inform dialogue about the MSK contribution to NCDs in children and the need for strategies to include a life course approach. In the absence of robust population based studies from many parts of the world, our work demonstrates many are living with MSK conditions, mostly in Asia and Africa and reflecting regions with high populations.

This information is important as the regions with highest estimates of children affected do not have robust population studies and many of the countries are LRIC. Consequently, it is likely that many affected children have little or no access to specialist care. Recent evidence reports worse clinical outcomes in JIA for countries with lower Gross Domestic Product [16]; it is possible that the same will be true for JSLE and clubfoot. JSLE remains a major cause of mortality in many parts of the world with late presentation and lack of treatments being available [18, 29, 30]. MSK conditions may be less prevalent than other NCDs such as diabetes [31] or inflammatory bowel disease [32], but the key message for public health systems is that the selected MSK conditions *are treatable and disability can be largely avoided.* In the case of clubfoot, the interventions is largely through physiotherapy at relatively low cost and significant benefit [11] and for many rheumatic diseases significant improvement can be observed with immunosuppressive treatments [13].

To address unmet need, there are many challenges to consider including awareness, workforce capacity, models of health care, and funding [18, 30, 33–35]. The WHO Global Burden Disability 2018 report [1] describes the health care workforce shortage in many parts of the world as being critical, more so in Asia and Africa and for the care of children [36]. Such parts of the world are most vulnerable to the burden of MSK disease given more ‘pressing’ health care need to address poverty, infection and malnutrition in health care resource limited environments.

There are several limitations in our study. We based our estimates on available reported prevalence rates for each condition and assumed stable prevalence over time. There is a spectrum of prevalence rates for JIA largely from Caucasian populations [15]; we used an average prevalence of 1 in 1000 and this may not be representative in terms of population or demographics (age stratification, gender or ethnicity) of individual countries. We did not attempt to address the reported variation in disease phenotype across racial groups in JIA [16, 17] or JSLE [25] or clubfoot [9]. Additionally, our calculations were subject to rounding error. Despite these limitations, we consider the data to be pragmatic estimates of the selected conditions and if anything, likely to be an underestimate. Comparison with historical population datasets for JIA from the UK [37] and Scandinavia [20] suggest that our estimates are reasonable, at least for countries with predominantly Caucasian populations. Our work highlights need for robust epidemiological data, especially from Africa and Asia, to inform dialogue about MSK burden in children and to include other MSK conditions such as Slipped Capital Femoral Epiphysis (SCFE) [38] where there is evidence of delay and early (surgical) intervention to improve clinical outcomes.

In conclusion, our work suggests that there are many children and young people with the selected MSK conditions around the world and many in regions challenged by limited resources and other health care problems. The Paediatric Global MSK Task Force [39] aims to raise awareness about the inequity in access to right care and a timely need for intervention. We hope that this study will facilitate dialogue with key stakeholders to leverage action.

## Supplementary information

**Additional file 1: Figures 4-6.** Estimated number of children and young people in 2017 living with clubfoot, JIA or JSLE respectively, within each sub-region. **Figure 4.** Estimated cases of clubfoot in 2017 by UN sub-region. **Figure 5.** Estimated cases of JIA in 2017 by UN sub-region. **Figure 6.** Estimated cases of JSLE in 2017 by UN sub-region. **Appendix A** - Country list by UN region and sub-region as used in our analysis (2017 data). **Appendix B** - Prevalence of clubfoot, JIA and JSLE by UN region and sub-region. **Table 1.** Estimated prevalence of clubfoot, JIA and JSLE by UN region in 2017, sorted in descending order of total population in 2017. **Table 2.** Estimated prevalence of clubfoot, JIA and JSLE by UN sub-region in 2017, sorted in descending order of total population in 2017. **Appendix C** - Estimated prevalence of clubfoot, JIA and JSLE by country in 2017, sorted in descending order of total population in 2017.

## Data Availability

All data generated or analysed during this study are included in this published article [and its supplementary information files].

## Abbreviations

CYP: Children and young people
DALYs: Disability adjusted life years
JIA: Juvenile Idiopathic Arthritis
JSLE: Juvenile Systemic Lupus Erythematosus
LRIC: Low Resource Income Countries
MRIC: Middle Resource Income Countries
MSK: Musculoskeletal
NCD: Non communicable diseases
SCFE: Slipped Capital Femoral Epiphysis
UK: United Kingdom
UN: United Nations
US: United States
WBG: World Bank Group
WHO: World Health Organisation
