# Supplementary material for: Global prevalence estimates of three chronic musculoskeletal conditions: club foot, juvenile idiopathic arthritis and juvenile systemic lupus erythematosus
Source: Pediatr Rheumatol Online J. 2020 Jun 12;18:49. doi: 10.1186/s12969-020-00443-8 (PMC7291758; doi:10.1186/s12969-020-00443-8)
Supplement: Supplementary file 1 — Additional file 1: Figures 4-6. Estimated number of children and young people in 2017 living with clubfoot, JIA or JSLE respectively, within each sub-region. Figure 4. Estimated cases of clubfoot in 2017 by UN sub-region. Figure 5. Estimated cases of JIA in 2017 by UN sub-region. Figure 6. Estimated cases of JSLE in 2017 by UN sub-region. Appendix A - Country list by UN region and sub-region as used in our analysis (2017 data). Appendix B - Prevalence of clubfoot, JIA and JSLE by UN region and sub-region. Table 1. Estimated prevalence of clubfoot, JIA and JSLE by UN region in 2017, sorted in descending order of total population in 2017. Table 2. Estimated prevalence of clubfoot, JIA and JSLE by UN sub-region in 2017, sorted in descending order of total population in 2017. Appendix C - Estimated prevalence of clubfoot, JIA and JSLE by country in 2017, sorted in descending order of total population in 2017. [file 12969_2020_443_MOESM1_ESM.docx]

**Figures 4-6: Estimated number of children and young people in 2017 living with clubfoot, JIA or JSLE respectively, within each sub-region**.

***Figure 4: Estimated cases of clubfoot in 2017 by UN sub-region***

**Figure 5: Estimated cases of JIA in 2017 by UN sub-region**

**Figure 6: Estimated cases of JSLE in 2017 by UN sub-region**

**Appendix A–Country list by UN region and sub-region as used in our analysis (2017 data)**

| **Africa** |  |  |  |  |
| --- | --- | --- | --- | --- |
| **Eastern Africa** | **Middle Africa** | **Northern Africa** | **Southern Africa** | **Western Africa** |
| Burundi  Comoros  Djibouti  Ethiopia  Kenya  Madagascar  Malawi  Mauritius  Mozambique  Rwanda  Seychelles  Somalia  South Sudan  Tanzania  Uganda  Zambia  Zimbabwe | Angola  Cameroon  Central African Republic  Chad  Congo, Democratic Republic  Congo, Republic | Algeria  Egypt, Arab Rep.  Libya  Morocco  Sudan  Tunisia | Botswana  Eswatini  Lesotho  Namibia  South Africa | Benin  Burkina Faso  Cabo Verde  Cote d’Ivoire  Gambia, The  Ghana  Guinea  Guinea-Bissau  Liberia  Mali  Mauritania  Niger  Nigeria  Senegal  Sierra Leone  Togo |
| **Americas** |  |  |  |  |
| **Caribbean** | **Central America** | **Northern America** | **South America** |  |
| Antigua and Barbuda  Aruba  Bahamas, The  Barbados  Cuba  Curacao  Dominican Republic  Grenada  Haiti  Jamaica  Puerto Rico  St. Lucia  St. Vincent and the Grenadines  Trinidad and Tobago  Virgin Islands (U.S.) | Belize  Costa Rica  El Salvador  Guatemala  Honduras  Mexico  Nicaragua  Panama | Canada  United States | Argentina  Bolivia  Brazil  Chile  Columbia  Ecuador  Guyana  Paraguay  Peru  Suriname  Uruguay  Venezuela, RB |  |
| **Asia** |  |  |  |  |
| **Central Asia** | **Eastern Asia** | **South-Eastern Asia** | **Southern Asia** | **Western Asia** |
| Kazakhstan  Kyrgyz Republic  Tajikistan  Turkmenistan  Uzbekistan | China  Hong Kong Special Administrative Region of the People’s Republic of China  Democratic People’s Republic of Korea  Republic of Korea  Macao Special Administrative Region of the People's Republic of China  Mongolia  Japan | Brunei Darussalam  Cambodia  Indonesia  Lao PDR  Malaysia  Myanmar  Philippines  Singapore  Thailand  Timor-Leste  Vietnam | Afghanistan  Bangladesh  Bhutan  India  Islamic Republic of Iran .  Maldives  Nepal  Pakistan  Sri Lanka | Armenia  Azerbaijan  Bahrain  Georgia  Iraq  Israel  Jordan  Kuwait  Lebanon  Oman  Qatar  Saudi Arabia  Syrian Arab Republic  United Arab Emirates  Republic of Yemen |
| **Europe** |  |  |  |  |
| **Eastern Europe** | **Northern Europe** | **Southern Europe** | **Western Europe** |  |
| Belarus  Bulgaria  Czech Republic  Hungary  Moldova  Poland  Romania  Russian Federation  Slovak Republic  Ukraine | Channel Islands  Denmark  Estonia  Finland  Iceland  Ireland  Latvia  Lithuania  Norway  Sweden  United Kingdom | Albania  Bosnia and Herzegovina  Croatia  Cyprus  Greece  Italy  Former Yugoslav Republic of Macedonia  Malta  Montenegro  Portugal  Serbia  Slovenia  Spain  Turkey | Austria  Belgium  France  Germany  Luxembourg  Netherlands  Switzerland |  |
| **Oceania** |  |  |  |  |
| **Australia and New Zealand** | **Melanesia** | **Micronesia** | **Polynesia** |  |
| Australia  New Zealand | Fiji  New Caledonia  Papua New Guinea  Solomon Islands  Vanuatu | Guam  Kiribati  Federated States of Micronesia | French Polynesia  Samoa  Tonga |  |

**Appendix B - Prevalence of clubfoot, JIA and JSLE by UN region and sub-region**

**Table 1: Estimated prevalence of clubfoot, JIA and JSLE by UN region in 2017, sorted in descending order of total population in 2017**

| **UN Region** | **Estimated number of <5 year olds living with clubfoot in 2017** | **Population <5 years old, 2017** | **Estimated number of <16 year olds living with JIA in 2017** | **Estimated number of <16 year olds living with JSLE in 2017** | **Population <16 years old, 2017** | **Total Population, 2017** |
| --- | --- | --- | --- | --- | --- | --- |
| Asia | 357,604 | 358,000,000 | 1,131,765 | 113,176 | 1,130,000,000 | 4,370,000,000 |
| Africa | 192,970 | 193,000,000 | 537,315 | 53,731 | 537,000,000 | 1,250,000,000 |
| Americas | 74,932 | 74,900,000 | 243,154 | 24,315 | 243,000,000 | 1,010,000,000 |
| Europe | 46,312 | 46,300,000 | 146,931 | 14,693 | 147,000,000 | 826,000,000 |
| Oceania | 3,243 | 3,243,144 | 10,078 | 1,008 | 10,100,000 | 40,700,000 |

**Table 2: Estimated prevalence of clubfoot, JIA and JSLE by UN sub-region in 2017, sorted in descending order of total population in 2017**

| **UN Sub-region** | **Estimated number of <5 year olds living with clubfoot in 2017** | **Population <5 years old, 2017** | **Estimated number of <16 year olds living with JIA in 2017** | **Estimated number of <16 year olds living with JSLE in 2017** | **Population <16 years old, 2017** | **Total Population, 2017** |
| --- | --- | --- | --- | --- | --- | --- |
| Southern Asia | 176,462 | 176,000,000 | 571,572 | 57,157 | 572,000,000 | 1,870,000,000 |
| Eastern Asia | 95,085 | 95,100,000 | 297,333 | 29,733 | 297,000,000 | 1,600,000,000 |
| South-Eastern Asia | 57,966 | 58,000,000 | 181,359 | 18,136 | 181,000,000 | 649,000,000 |
| South America | 33,271 | 33,300,000 | 107,646 | 10,765 | 108,000,000 | 424,000,000 |
| Eastern Africa | 66,158 | 66,200,000 | 187,787 | 18,779 | 188,000,000 | 416,000,000 |
| Western Africa | 62,355 | 62,400,000 | 171,062 | 17,106 | 171,000,000 | 372,000,000 |
| Northern America | 21,660 | 21,700,000 | 71,799 | 7,180 | 71,800,000 | 362,000,000 |
| Eastern Europe | 16,888 | 16,900,000 | 50,673 | 5,067 | 50,700,000 | 293,000,000 |
| Northern Africa | 28,776 | 28,800,000 | 79,760 | 7,976 | 79,800,000 | 233,000,000 |
| Southern Europe | 13,408 | 13,400,000 | 44,946 | 4,495 | 44,900,000 | 233,000,000 |
| Western Europe | 9,852 | 9,852,480 | 31,874 | 3,187 | 31,900,000 | 196,000,000 |
| Western Asia | 20,276 | 20,300,000 | 59,572 | 5,957 | 59,600,000 | 181,000,000 |
| Central America | 16,480 | 16,500,000 | 52,480 | 5,248 | 52,500,000 | 177,000,000 |
| Middle Africa | 28,891 | 28,900,000 | 78,112 | 7,811 | 78,100,000 | 163,000,000 |
| Northern Europe | 6,162 | 6,162,235 | 19,438 | 1,944 | 19,400,000 | 104,000,000 |
| Central Asia | 7,814 | 7,814,271 | 21,928 | 2,193 | 21,900,000 | 71,300,000 |
| Southern Africa | 6,790 | 6,790,434 | 20,593 | 2,059 | 20,600,000 | 65,100,000 |
| Caribbean | 3,521 | 3,521,354 | 11,230 | 1,123 | 11,200,000 | 42,400,000 |
| Australia and New Zealand | 1,878 | 1,878,097 | 5,928 | 593 | 5,927,596 | 29,400,000 |
| Melanesia | 1,267 | 1,267,434 | 3,838 | 384 | 3,838,366 | 10,300,000 |
| Polynesia | 58 | 57,789 | 188 | 19 | 187,882 | 587,467 |
| Micronesia | 40 | 39,824 | 124 | 12 | 123,658 | 386,171 |

**Appendix C - Estimated prevalence of clubfoot, JIA and JSLE by country in 2017, sorted in descending order of total population in 2017**

| **Country Name** | **Estimated number of <5 year olds living with clubfoot in 2017 (N)** | **Population <5 years old (N)** | **Estimated number of <16 year olds living with JIA in 2017 (N)** | **Estimated number of <16 year olds living with JSLE in 2017 (N)** | **Population <16 years old (N)** | **Total Population (N)** |
| --- | --- | --- | --- | --- | --- | --- |
| China | 85,131 | 85,100,000 | 264,792 | 26,479 | 265,000,000 | 1,386,000,000 |
| India | 119,757 | 120,000,000 | 397,283 | 39,728 | 397,000,000 | 1,339,000,000 |
| United States | 19,724 | 19,700,000 | 65,548 | 6,555 | 65,500,000 | 325,700,000 |
| Indonesia | 24,725 | 24,700,000 | 76,929 | 7,693 | 76,900,000 | 264,000,000 |
| Brazil | 14,828 | 14,800,000 | 48,836 | 4,884 | 48,800,000 | 209,300,000 |
| Pakistan | 25,122 | 25,100,000 | 72,341 | 7,234 | 72,300,000 | 197,000,000 |
| Nigeria | 32,378 | 32,400,000 | 88,257 | 8,826 | 88,300,000 | 190,900,000 |
| Bangladesh | 15,167 | 15,200,000 | 49,999 | 5,000 | 50,000,000 | 164,700,000 |
| Russian Federation | 9,527 | 9,527,043 | 26,704 | 2,670 | 26,700,000 | 144,500,000 |
| Mexico | 11,547 | 11,500,000 | 36,782 | 3,678 | 36,800,000 | 129,200,000 |
| Japan | 5,293 | 5,293,120 | 17,572 | 1,757 | 17,600,000 | 126,800,000 |
| Ethiopia | 15,366 | 15,400,000 | 45,124 | 4,512 | 45,100,000 | 105,000,000 |
| Philippines | 11,611 | 11,600,000 | 35,344 | 3,534 | 35,300,000 | 104,900,000 |
| Egypt, Arab Rep. | 12,918 | 12,900,000 | 34,400 | 3,440 | 34,400,000 | 97,553,151 |
| Vietnam | 7,745 | 7,745,454 | 23,339 | 2,334 | 23,300,000 | 95,540,800 |
| Germany | 3,595 | 3,595,121 | 11,499 | 1,150 | 11,500,000 | 82,695,000 |
| Congo, Dem. Rep. | 14,846 | 14,800,000 | 39,471 | 3,947 | 39,500,000 | 81,339,988 |
| Iran, Islamic Rep. | 6,716 | 6,715,660 | 20,278 | 2,028 | 20,300,000 | 81,162,788 |
| Turkey | 6,728 | 6,728,065 | 21,502 | 2,150 | 21,500,000 | 80,745,020 |
| Thailand | 3,679 | 3,678,586 | 12,847 | 1,285 | 12,800,000 | 69,037,513 |
| France | 3,824 | 3,824,180 | 12,533 | 1,253 | 12,500,000 | 67,118,648 |
| United Kingdom | 3,999 | 3,999,261 | 12,426 | 1,243 | 12,400,000 | 66,022,273 |
| Italy | 2,448 | 2,447,603 | 8,585 | 858 | 8,584,742 | 60,551,416 |
| Tanzania | 9,862 | 9,862,423 | 27,053 | 2,705 | 27,100,000 | 57,310,019 |
| South Africa | 5,712 | 5,712,002 | 17,470 | 1,747 | 17,500,000 | 56,717,156 |
| Myanmar | 4,524 | 4,524,014 | 15,362 | 1,536 | 15,400,000 | 53,370,609 |
| Korea, Rep. | 2,230 | 2,229,755 | 7,390 | 739 | 7,389,742 | 51,466,201 |
| Kenya | 7,099 | 7,098,564 | 21,258 | 2,126 | 21,300,000 | 49,699,862 |
| Colombia | 3,682 | 3,682,272 | 12,336 | 1,234 | 12,300,000 | 49,065,615 |
| Spain | 2,017 | 2,016,865 | 7,256 | 726 | 7,256,272 | 46,572,028 |
| Ukraine | 2,303 | 2,303,047 | 7,227 | 723 | 7,226,690 | 44,831,159 |
| Argentina | 3,737 | 3,737,484 | 11,730 | 1,173 | 11,700,000 | 44,271,041 |
| Uganda | 7,882 | 7,881,615 | 21,492 | 2,149 | 21,500,000 | 42,862,958 |
| Algeria | 4,663 | 4,662,792 | 12,657 | 1,266 | 12,700,000 | 41,318,142 |
| Sudan | 6,021 | 6,021,330 | 17,486 | 1,749 | 17,500,000 | 40,533,330 |
| Iraq | 5,849 | 5,848,752 | 16,283 | 1,628 | 16,300,000 | 38,274,618 |
| Poland | 1,774 | 1,774,070 | 6,008 | 601 | 6,008,064 | 37,975,841 |
| Canada | 1,936 | 1,936,073 | 6,251 | 625 | 6,250,512 | 36,708,083 |
| Morocco | 3,502 | 3,501,520 | 10,387 | 1,039 | 10,400,000 | 35,739,580 |
| Afghanistan | 5,271 | 5,271,257 | 16,264 | 1,626 | 16,300,000 | 35,530,081 |
| Saudi Arabia | 2,990 | 2,990,050 | 8,755 | 876 | 8,755,159 | 32,938,213 |
| Uzbekistan | 3,180 | 3,180,210 | 9,431 | 943 | 9,430,824 | 32,387,200 |
| Peru | 3,027 | 3,026,860 | 9,369 | 937 | 9,369,237 | 32,165,485 |
| Venezuela | 2,971 | 2,971,313 | 9,393 | 939 | 9,392,901 | 31,977,065 |
| Malaysia | 2,644 | 2,643,663 | 8,223 | 822 | 8,223,033 | 31,624,264 |
| Angola | 5,400 | 5,400,401 | 14,624 | 1,462 | 14,600,000 | 29,784,193 |
| Mozambique | 5,047 | 5,047,300 | 13,986 | 1,399 | 14,000,000 | 29,668,834 |
| Nepal | 2,745 | 2,745,435 | 9,726 | 973 | 9,725,938 | 29,304,998 |
| Ghana | 4,124 | 4,124,045 | 11,716 | 1,172 | 11,700,000 | 28,833,629 |
| Yemen, Rep. | 4,114 | 4,114,449 | 11,916 | 1,192 | 11,900,000 | 28,250,420 |
| Madagascar | 3,847 | 3,847,427 | 11,069 | 1,107 | 11,100,000 | 25,570,895 |
| Korea, Democratic Peoples Republic | 1,733 | 1,733,375 | 5,638 | 564 | 5,637,922 | 25,490,965 |
| Australia | 1,574 | 1,574,104 | 4,938 | 494 | 4,937,848 | 24,598,933 |
| Cote d'Ivoire | 3,947 | 3,946,744 | 10,864 | 1,086 | 10,900,000 | 24,294,750 |
| Cameroon | 3,861 | 3,860,517 | 10,818 | 1,082 | 10,800,000 | 24,053,727 |
| Niger | 4,366 | 4,366,441 | 11,285 | 1,128 | 11,300,000 | 21,477,348 |
| Sri Lanka | 1,575 | 1,575,140 | 5,346 | 535 | 5,345,719 | 21,444,000 |
| Romania | 932 | 931,815 | 3,211 | 321 | 3,211,453 | 19,586,539 |
| Burkina Faso | 3,282 | 3,281,716 | 9,120 | 912 | 9,120,303 | 19,193,382 |
| Malawi | 2,966 | 2,966,272 | 8,631 | 863 | 8,631,246 | 18,622,104 |
| Mali | 3,396 | 3,395,595 | 9,280 | 928 | 9,280,065 | 18,541,980 |
| Syrian Arab Republic | 1,979 | 1,979,354 | 7,139 | 714 | 7,139,498 | 18,269,868 |
| Chile | 1,179 | 1,178,697 | 3,922 | 392 | 3,922,273 | 18,054,726 |
| Kazakhstan | 1,970 | 1,970,483 | 5,297 | 530 | 5,296,534 | 18,037,646 |
| Netherlands | 897 | 896,707 | 3,001 | 300 | 3,001,183 | 17,132,854 |
| Zambia | 2,889 | 2,888,526 | 8,070 | 807 | 8,070,129 | 17,094,130 |
| Guatemala | 2,040 | 2,039,671 | 6,319 | 632 | 6,318,824 | 16,913,503 |
| Ecuador | 1,613 | 1,612,872 | 5,027 | 503 | 5,026,671 | 16,624,858 |
| Zimbabwe | 2,549 | 2,548,654 | 7,171 | 717 | 7,170,684 | 16,529,904 |
| Cambodia | 1,766 | 1,766,004 | 5,297 | 530 | 5,297,111 | 16,005,373 |
| Senegal | 2,584 | 2,583,829 | 7,145 | 715 | 7,145,006 | 15,850,567 |
| Chad | 2,726 | 2,725,997 | 7,383 | 738 | 7,383,139 | 14,899,994 |
| Somalia | 2,686 | 2,686,126 | 7,188 | 719 | 7,187,865 | 14,742,523 |
| Guinea | 2,017 | 2,016,937 | 5,662 | 566 | 5,662,281 | 12,717,176 |
| South Sudan | 1,966 | 1,966,242 | 5,526 | 553 | 5,526,491 | 12,575,714 |
| Rwanda | 1,749 | 1,748,806 | 5,170 | 517 | 5,169,945 | 12,208,407 |
| Tunisia | 1,053 | 1,052,953 | 2,925 | 293 | 2,925,484 | 11,532,127 |
| Cuba | 633 | 632,591 | 1,969 | 197 | 1,969,384 | 11,484,636 |
| Belgium | 647 | 646,508 | 2,079 | 208 | 2,078,852 | 11,372,068 |
| Benin | 1,809 | 1,809,240 | 5,021 | 502 | 5,020,734 | 11,175,692 |
| Bolivia | 1,194 | 1,193,852 | 3,721 | 372 | 3,720,747 | 11,051,600 |
| Haiti | 1,232 | 1,232,274 | 3,849 | 385 | 3,848,809 | 10,981,229 |
| Burundi | 1,952 | 1,952,135 | 5,116 | 512 | 5,116,135 | 10,864,245 |
| Dominican Republic | 1,055 | 1,055,374 | 3,358 | 336 | 3,357,520 | 10,766,998 |
| Greece | 458 | 457,989 | 1,695 | 169 | 1,694,931 | 10,760,421 |
| Czech Republic | 529 | 529,111 | 1,725 | 173 | 1,725,190 | 10,591,323 |
| Portugal | 419 | 418,988 | 1,515 | 152 | 1,515,256 | 10,293,718 |
| Sweden | 590 | 589,971 | 1,841 | 184 | 1,841,331 | 10,067,744 |
| Azerbaijan | 885 | 884,686 | 2,415 | 241 | 2,414,652 | 9,862,429 |
| Hungary | 433 | 432,622 | 1,489 | 149 | 1,488,742 | 9,781,127 |
| Jordan | 1,240 | 1,239,617 | 3,642 | 364 | 3,642,388 | 9,702,353 |
| Belarus | 577 | 577,063 | 1,672 | 167 | 1,671,822 | 9,507,875 |
| United Arab Emirates | 457 | 457,061 | 1,378 | 138 | 1,377,929 | 9,400,145 |
| Honduras | 953 | 953,105 | 3,134 | 313 | 3,134,139 | 9,265,067 |
| Tajikistan | 1,194 | 1,194,241 | 3,308 | 331 | 3,308,258 | 8,921,343 |
| Austria | 416 | 416,381 | 1,316 | 132 | 1,315,917 | 8,809,212 |
| Israel | 842 | 841,580 | 2,451 | 245 | 2,450,609 | 8,712,400 |
| Switzerland | 440 | 440,445 | 1,343 | 134 | 1,343,370 | 8,466,017 |
| Papua New Guinea | 1,043 | 1,042,547 | 3,143 | 314 | 3,142,863 | 8,251,162 |
| Togo | 1,190 | 1,190,096 | 3,417 | 342 | 3,416,852 | 7,797,694 |
| Sierra Leone | 1,150 | 1,149,900 | 3,357 | 336 | 3,357,143 | 7,557,212 |
| Hong Kong SAR, China | 296 | 295,741 | 900 | 90 | 899,548 | 7,391,700 |
| Bulgaria | 320 | 320,465 | 1,070 | 107 | 1,069,658 | 7,075,991 |
| Serbia | 467 | 467,329 | 1,552 | 155 | 1,552,269 | 7,022,268 |
| Lao Peoples Democratic Republic | 763 | 763,420 | 2,396 | 240 | 2,396,166 | 6,858,160 |
| Paraguay | 674 | 673,857 | 2,133 | 213 | 2,133,314 | 6,811,297 |
| El Salvador | 575 | 575,068 | 1,868 | 187 | 1,868,218 | 6,377,853 |
| Libya | 619 | 619,239 | 1,904 | 190 | 1,904,109 | 6,374,616 |
| Nicaragua | 592 | 591,697 | 1,924 | 192 | 1,923,740 | 6,217,581 |
| Kyrgyz Republic | 757 | 756,851 | 2,016 | 202 | 2,016,435 | 6,201,500 |
| Lebanon | 489 | 488,922 | 1,509 | 151 | 1,509,309 | 6,082,357 |
| Denmark | 285 | 285,455 | 1,012 | 101 | 1,012,136 | 5,769,603 |
| Turkmenistan | 712 | 712,486 | 1,876 | 188 | 1,876,138 | 5,758,075 |
| Singapore | 266 | 266,158 | 921 | 92 | 921,181 | 5,612,253 |
| Finland | 297 | 297,473 | 964 | 96 | 964,480 | 5,511,303 |
| Slovak Republic | 280 | 280,147 | 889 | 89 | 889,486 | 5,439,892 |
| Norway | 309 | 308,752 | 1,006 | 101 | 1,005,658 | 5,282,223 |
| Congo, Rep. | 833 | 832,674 | 2,337 | 234 | 2,337,066 | 5,260,750 |
| Costa Rica | 343 | 343,374 | 1,135 | 113 | 1,134,882 | 4,905,769 |
| Ireland | 338 | 337,788 | 1,091 | 109 | 1,090,792 | 4,813,608 |
| New Zealand | 304 | 303,993 | 990 | 99 | 989,748 | 4,793,900 |
| Liberia | 727 | 727,170 | 2,088 | 209 | 2,087,745 | 4,731,906 |
| Central African Republic | 730 | 730,414 | 2,128 | 213 | 2,127,541 | 4,659,080 |
| Oman | 407 | 407,420 | 1,057 | 106 | 1,056,732 | 4,636,262 |
| Mauritania | 667 | 667,225 | 1,860 | 186 | 1,859,666 | 4,420,184 |
| Kuwait | 320 | 320,416 | 918 | 92 | 918,363 | 4,136,528 |
| Croatia | 192 | 191,604 | 656 | 66 | 656,201 | 4,125,700 |
| Panama | 389 | 389,029 | 1,193 | 119 | 1,192,731 | 4,098,587 |
| Georgia | 266 | 265,853 | 792 | 79 | 791,859 | 3,717,100 |
| Moldova | 213 | 213,070 | 678 | 68 | 677,612 | 3,549,750 |
| Bosnia and Herzegovina | 155 | 155,329 | 532 | 53 | 531,560 | 3,507,017 |
| Uruguay | 239 | 238,829 | 778 | 78 | 777,980 | 3,456,750 |
| Puerto Rico | 195 | 194,885 | 708 | 71 | 707,914 | 3,337,177 |
| Mongolia | 366 | 365,806 | 955 | 95 | 954,543 | 3,075,647 |
| Armenia | 198 | 198,244 | 620 | 62 | 619,930 | 2,930,450 |
| Jamaica | 209 | 209,288 | 706 | 71 | 705,596 | 2,890,299 |
| Albania | 177 | 177,179 | 551 | 55 | 550,728 | 2,873,457 |
| Lithuania | 152 | 151,913 | 456 | 46 | 455,983 | 2,827,721 |
| Qatar | 132 | 132,329 | 387 | 39 | 386,588 | 2,639,211 |
| Namibia | 349 | 348,513 | 984 | 98 | 983,677 | 2,533,794 |
| Botswana | 260 | 260,108 | 762 | 76 | 762,474 | 2,291,661 |
| Lesotho | 289 | 288,675 | 839 | 84 | 838,984 | 2,233,339 |
| Gambia, The | 368 | 367,853 | 1,001 | 100 | 1,001,064 | 2,100,568 |
| Macedonia | 118 | 118,346 | 372 | 37 | 372,401 | 2,083,160 |
| Slovenia | 106 | 106,350 | 329 | 33 | 329,141 | 2,066,748 |
| Gabon | 277 | 277,204 | 765 | 77 | 765,271 | 2,025,137 |
| Latvia | 95 | 94,977 | 318 | 32 | 317,944 | 1,940,740 |
| Guinea-Bissau | 295 | 295,029 | 813 | 81 | 812,612 | 1,861,283 |
| Bahrain | 108 | 107,586 | 311 | 31 | 310,629 | 1,492,584 |
| Trinidad and Tobago | 93 | 92,794 | 301 | 30 | 300,519 | 1,369,125 |
| Eswatini | 181 | 181,136 | 538 | 54 | 538,318 | 1,367,254 |
| Estonia | 67 | 66,958 | 227 | 23 | 226,537 | 1,315,480 |
| Timor-Leste | 208 | 208,053 | 596 | 60 | 596,035 | 1,296,311 |
| Equatorial Guinea | 187 | 186,622 | 495 | 49 | 494,600 | 1,267,689 |
| Mauritius | 67 | 67,437 | 252 | 25 | 251,958 | 1,264,613 |
| Cyprus | 65 | 65,373 | 212 | 21 | 212,464 | 1,179,551 |
| Djibouti | 103 | 102,625 | 317 | 32 | 317,272 | 956,985 |
| Fiji | 86 | 85,640 | 273 | 27 | 273,405 | 905,502 |
| Comoros | 121 | 120,533 | 341 | 34 | 341,380 | 813,912 |
| Bhutan | 70 | 69,688 | 229 | 23 | 228,860 | 807,610 |
| Guyana | 77 | 76,611 | 242 | 24 | 241,694 | 777,859 |
| Macao SAR*, China | 36 | 35,958 | 87 | 9 | 86,764 | 622,567 |
| Montenegro | 36 | 35,779 | 122 | 12 | 121,815 | 622,471 |
| Solomon Islands | 83 | 82,877 | 251 | 25 | 251,248 | 611,343 |
| Luxembourg | 33 | 33,138 | 103 | 10 | 102,596 | 599,449 |
| Suriname | 50 | 50,195 | 159 | 16 | 158,765 | 563,402 |
| Cabo Verde | 55 | 54,713 | 176 | 18 | 176,232 | 546,388 |
| Malta | 22 | 21,574 | 66 | 7 | 66,300 | 465,292 |
| Maldives | 39 | 39,146 | 108 | 11 | 107,567 | 436,330 |
| Brunei Darussalam | 34 | 34,358 | 106 | 11 | 105,621 | 428,697 |
| Bahamas, The | 27 | 27,488 | 86 | 9 | 85,901 | 395,361 |
| Belize | 41 | 40,879 | 126 | 13 | 125,691 | 374,681 |
| Iceland | 22 | 21,880 | 72 | 7 | 71,520 | 341,284 |
| Barbados | 17 | 17,223 | 58 | 6 | 58,234 | 285,719 |
| French Polynesia | 22 | 22,182 | 71 | 7 | 70,528 | 283,007 |
| New Caledonia | 22 | 21,709 | 66 | 7 | 66,324 | 280,460 |
| Vanuatu | 35 | 34,661 | 105 | 10 | 104,526 | 276,244 |
| Sao Tome and Principe | 32 | 31,599 | 92 | 9 | 92,435 | 204,327 |
| Samoa | 23 | 23,060 | 76 | 8 | 76,159 | 196,440 |
| St. Lucia | 11 | 10,920 | 37 | 4 | 36,550 | 178,844 |
| Channel Islands | 8 | 7,807 | 26 | 3 | 25,703 | 165,314 |
| Guam | 14 | 13,551 | 43 | 4 | 43,434 | 164,229 |
| Curacao | 10 | 10,347 | 32 | 3 | 32,101 | 161,014 |
| Kiribati | 15 | 14,542 | 43 | 4 | 42,788 | 116,398 |
| St. Vincent and the Grenadines | 8 | 8,183 | 28 | 3 | 27,970 | 109,897 |
| Tonga | 13 | 12,547 | 41 | 4 | 41,195 | 108,020 |
| Grenada | 10 | 9,808 | 30 | 3 | 30,145 | 107,825 |
| Virgin Islands (U.S.) | 7 | 6,706 | 22 | 2 | 22,485 | 107,268 |
| Micronesia, Fed. Sts. | 12 | 11,731 | 37 | 4 | 37,436 | 105,544 |
| Aruba | 5 | 5,394 | 20 | 2 | 20,303 | 105,264 |
| Antigua and Barbuda | 8 | 8,079 | 26 | 3 | 26,071 | 102,012 |
| Seychelles | 8 | 7,753 | 22 | 2 | 22,193 | 95,843 |

*Hong Kong SAR Special Administrative Region of the People’s Republic of China *Macao SAR (Special Administrative Region) of the People's Republic of China
